# The Prediction of Public Risk Perception by Internal Characteristics and External Environment: Machine Learning on Big Data

**DOI:** 10.3390/ijerph19159545

**Published:** 2022-08-03

**Authors:** Qihui Xie, Yanan Xue

**Affiliations:** Department of Public Administration, School of Law and Humanities, China University of Mining and Technology (Beijing), Beijing 100083, China; sqt2000803033@student.cumtb.edu.cn

**Keywords:** prediction, public risk perception, big data, BP neural network, Sino–US trade friction, COVID-19 pandemic

## Abstract

Presently, the public’s perception of risk in terms of topical social issues is mainly measured quantitively using a psychological scale, but this approach is not accurate enough for everyday data. In this paper, we explored the ways in which public risk perception can be more accurately predicted in the era of big data. We obtained internal characteristics and external environment predictor variables through a literature review, and then built our prediction model using the machine learning of a BP neural network via three steps: the calculation of the node number of the implication level, a performance test of the BP neural network, and the computation of the weight of every input node. Taking the public risk perception of the Sino–US trade friction and the COVID-19 pandemic in China as research cases, we found that, according to our tests, the node number of the implication level was 15 in terms of the Sino–US trade friction and 14 in terms of the COVID-19 pandemic. Following this, machine learning was conducted, through which we found that the *R*^2^ of the BP neural network prediction model was 0.88651 and 0.87125, respectively, for the two cases, which accurately predicted the public’s risk perception of the data on a certain day, and simultaneously obtained the weight of every predictor variable in each case. In this paper, we provide comments and suggestions for building a model to predict the public’s perception of topical issues.

## 1. Introduction

As the availability of information increases to levels too large to manually intercept, manage, process, and organize within a rational timescale due to the Internet and information clustering, the concept of “big data” thus emerges [1]. In addition to the large volume, big data also involve issues relating to veracity [2]. Since big data come from human activities, people’s attitude and behavior can be reflected by the analysis of big data; therefore, big data analysis is widely applied in the psychological and behavioral analysis of humans, and risk perception is one of the recognized facets of human psychology. Public risk perception, especially towards topical issues or crisis scenarios, is an important indicator of individuals’ psychological health. In the era of big data, understanding how to obtain effective, scientific, and real-time predictions of risk perception is an important path to promote human health.

Research on risk perception originated from the understanding of the concept of risk. Risk refers to the possibility of an adverse event taking place. The understanding of the concept of risk has been divided since the 1950s, and involves different objective and subjective views. According to the objective view, there is no difference at the level of subjective cognition in terms of risk, but risk is a phenomenon that exists universally in post-modern society [3]. However, research scholars who take the subjective view have reached two consensuses on the concept of risk. 1. Risks vary along with changes in people’s cognition. For example, Douglas and Wildavsky (1983) [4] thought that risks did not increase or intensify in modern society; on the contrary, the conscious risks observed were found to both increase and intensify. Lash and Wang (2002) [5] thought that the perception of risk increased from the perspective of the subject’s consciousness enhancement. In this paper, the notion of risk perception—the core object under study—originated from the consensus: i.e., it is the subjective judgment of people concerning risks [6], namely, people’s assessment of the risks actually or potentially affecting them [7,8], for example, a risk’s possibility, control, and expectedness [9]. Another consensus reached by research scholars of subjective risk theory on the cognition of the risk concept is that people’s subjective judgment of risks may be influenced by social construction. From the perspective of Slovic (1987), a risk event might influence risk perception after passing through the process of social interpretation when analyzing the ripple effect caused by the risk event [7], and thereby the Social Amplification of Risk Framework (SARF) was proposed. Dake and Wildavsky (1991) posited that personal risk perception is influenced by public social values and ideology [10]. Such consensus provided us with the theoretical basis for the prediction of risk perception’s influence factors: risk perception is constructed by society, so we can effectively predict risk perception if we can identify the factors constructed by society.

Many scholars have researched risk perception and its influence factors. Many scholars verified the effective influence of various influence factors on risk perception using quantitative research methods [11,12], which provided literature-based support to the construction of a predictive model in this paper. Most of the measurements of risk perception in existing quantitative research focused on the psychological scale [13,14] and questionnaire method, both of which have been verified for their validity and effectiveness. However, there are certain problems with these approaches, namely, that the means of acquiring data is possibly too subjective, and there is no way to obtain dynamic measurements on a daily basis. In the current era of information, an increasing number of people prefer to express their intentions on the Internet, so mining and analyzing the big data of such intentions and their influence factors can help to measure the extent of the influence factors of risk perception more objectively and accurately, and even make predictions, but there is very little research available on this topic currently. In this paper, our research attempted to add to the literature on risk perception using big data and supplement the risk perception research using a traditional questionnaire method.

In the literature review, we first acquired the influence factors of two dimensions, namely, external environment and content characteristics, that are possibly involved in risk perception, and then selected the most appropriate method to measure and predict risk perception in the era of big data. Secondly, we explored the ways in which to acquire the data pertaining to public risk perception and their predictive factors using big data from a risk event before constructing the predictive model. Lastly, we analyzed two risk events related to the economic environment and public health environment, namely, the “Sino–US Trade Friction” and the “COVID-19 pandemic”, using the BP neural network (i.e., machine learning) method, and then effectively predicted Chinese people’s risk perception of these two events. All of the results in the paper can help decision makers to effectively predict and cope with risk perception in terms of social risk events, and simultaneously provide an innovative method for realizing the dynamic prediction of the public’s risk perception in terms of a specific day.

## 2. Literature Review

### 2.1. Factors Influencing Risk Perception

In this paper, we built a prediction model of public risk perception based on the factors influencing risk perception. Whilst identifying the literature concerning risk perception, we found that there were many factors that have an influence, which not only affect risk perception independently, but also through a cross-action effect. In general, the factors influencing risk perception according to the existing studies can be divided into two categories: the internal characteristics of a public group, and the external environmental influence on a public group. 

#### 2.1.1. Internal Characteristics 

The factors that have an influence on a public group’s internal characteristics for risk perception include three types: personal characteristics, risk experience, and cultural and economic characteristics.

1. Personal Characteristics

The factors influencing public risk perception that are analyzed from the dimension of personal characteristics include age, gender, education level, annual income, profession, residential community, belief, and ethnicity [15]. Among the existing studies, certain scholars asserted that there was significant correlation between personal characteristics and personal risk perception. For example, Seeger et al. (2010) [16] believed that the individual characteristics of the public were the direct reason for the differences in their attitude towards risk, and risk attitude directly affects the public risk perception. There are plenty of studies on this aspect. For example, Cutter (1992) [17] studied the differences in public risk perception among different genders, and found that females were more pessimistic than males. Lindell et al. (2008) [18] found that lower educational and income levels, being female, and being of a minority group or ethnicity with lower status might lead to individuals having higher risk perception. Hakes (2004) [19] posited that education could enable people to perceive risks more closely to objective fact, and people with older age might be more reasonable. Additionally, Sjöberg (2003) [20] and Siegrist (2020) [21] asserted that the aforementioned factors were slightly correlated with risk perception. Among many studies, personal characteristic factors, especially population characteristics, were frequently used as the control variables, as they do not have a decisive influence on the risk perception of the relevant subjects. However, in this paper, our purpose was not to verify influence factors, but to verify predictive factors, in order to put personal characteristics into the prediction model to investigate whether personal characteristic factors could predict risk perception or not.

2. Risk Experience

Risk experience refers to whether individuals have experienced a similar risk event or not. Most people might improve their risk perception of correlation, frequency, and hazard [22,23] after actually experiencing a risk event, making it easier for them to understand the controllability and seriousness of a risk, and thus become more likely to actively search for and understand the relevant risk information. People’s feelings of a situation being out of control might give rise to the improvement of personal risk perception. Other scholars assert that risk experience might be embodied as the accumulation of risk knowledge. For example, Mutz (1992) [24] believed that risk perception might be correlated with views on social problems. Additionally, certain scholars identified two factors, namely, scientific knowledge and personal knowledge, respectively as objective knowledge and the subjective knowledge, but both of these factors were collectively named “knowledge structure” by other scholars. The issue of how crisis knowledge affects public risk perception is in dispute. For example, Slovic (1981) [25] discussed how the difference in risk knowledge structure was the reason for the difference in risk perception, but Rowe et al. (2001) [26] disagreed with the viewpoint. Thus, in this paper, we incorporated whether people went through risks into our model, instead of incorporating the knowledge in dispute into our model.

3. Cultural and Economic Characteristics

Culture is deemed one of the most important factors affecting public risk perception, and the cultural characteristics of every social subject (such as the difference in culture and social background) decide the type of the risk people are concerned about [27]. An important research paradigm of risk perception, the sociocultural theory of risk, originated from the perspective of culture. Knight et al. (2010) [28] believed that the ideology (including political orientation, religion, and views of value) should also be covered by a cultural dimension, where personal perception and attitudes towards a certain issue do not emerge out of thin air, but are decided by personal socialization and personal belief systems for politics and society. Yang (2015) [29] found that power distance and collectivism were the cultural factors affecting the risk perception of Asians.

Economic factors are also deemed important factors influencing changes in risk perception [30]. Generally, economic factors mainly involve the public’s income level, including various variables such as monthly income, annual income, and household income. Gaillard (2008) [31], on the basis of natural disasters, conducted an analysis based upon personal economic characteristics and found that communities with limited or unstable income might be more vulnerable and have higher risk perception when faced with disasters. Zhang et al. (2021) [32], on the basis of a public health event, analyzed urban economy characteristics and reached the following conclusion: the higher the average wage in a region, the larger the population density, and the higher the risk perception of the public in the region. In typical analyses, economic factors are always combined with cultural factors, social composition factors, and other related factors [32]. In this paper, we combined cultural factors and economic factors into one factor and incorporate it into our predictive model.

#### 2.1.2. External Environment

1. Crisis Information

Certain scholars studied the influence of environmental factors on risk perception beyond public groups. Among the external environment factors, most studies focused on the influence of the information dimension. According to the results, the spread of crisis information promoted the formation of public risk perception [33], and worked as an important means of enabling the public to deal with a crisis as they may continue exploring and accumulating information, thereby making decisions based on avoiding risks and reducing the degree of threat from the crisis. For the analysis of the influence of the information dimension on risk perception, the factors include information amount, type, difference, and subject [34]. 

Information amount is the amplifier of risk; however, studies on the influence of information amount on risk perception are still in dispute in academic circles. For example, Renn and Rohrmann (2000) [3] posited that a shortage of information could lead to the high-risk perception of the public. However, Weinberg (1977) [35] asserted that too much information might intensify a crisis event and thereby result in an overestimate of the risk. 

Language is the carrier of information. Public risk perception tends to be heavily influenced by the speeches made regarding risk events. Through pictures, videos, and other similar information sources, a vivid presentation of an event can be provided, and more intense emotion and higher risk perception can be induced [36]. Moreover, differences in the information acquired by the public (such as frequency, type, etc.) may cause the public to respond to risks and crisis events differently [37]. 

According to most of the studies, the media should be considered and measured as the core element influencing public perception; the media may promote the spread of risk information under the risk amplification framework, thereby promoting the risk perception of the public [38]. Additionally, the factor of people’s trust in the information source is also frequently considered in the studies of risk perception. The evolution of public risk perception is somewhat subjective, and moreover, there is the phenomenon of information asymmetry between the public and the information source, where government, news media, and experts are the uppermost risk information sources and are trusted the most by the public [39]. Giddens (1990) [40] found a positive correlation between trust and the risk perception of a spreading subject. When the public’s knowledge is not sufficient to cope with a risk or crisis, they may possibly maintain their relative safety by trusting experts or scientists. Later, Vandermoere (2008) [41] further verified there was a positive correlation among experts’/scientists’ objective assessment of crisis fact, risk perception status, and public risk perception. This can also explain the reason why governments frequently convene higher-level expert forums and popularize scientific facts when coping with crises. On this basis, in this paper, we opted to incorporate medium information intervention and government information intervention into the prediction model.

2. Risk Characteristics

Generally, risk characteristics refer to the time characteristics of risk, namely, the possible influence on risk perception at different stages of risk development. Moser et al. (2012) [42] posited that risk’s temporal representation (including linear time and periodic time) might influence risk perception, where linear time refers to when a risk is irreversible and will not happen again once it has occurred, and periodic time describes risks that may happen repeatedly. According to the consensus from present studies, risk perception may reduce as the linear time of a crisis event increases. For example, according to Wei et al. (2012) [43], the public might forget the crisis information in accordance with the Ebbinghaus forgetting curve. However, studies on how periodic risk time characteristics vary with risk perception are few; therefore, there is much room for researching the influence of the periodic time factor on public risk perception. In order to increase studies of this type, we incorporated the periodic time factor into the prediction model in this paper. 

### 2.2. Big Data Measurement Method of Risk Perception

Existing studies acquired the data regarding public risk perception in crisis situations using static and microcosmic personal questionnaires [18,44]. However, this method has the shortcomings of being time consuming, hysteretic in nature, and costly. Thus, scholars began exploring ways of realizing dynamic and real-time monitoring of risk perception using cutting-edge big data technology, with two main methods being adopted. The first method is based on network searching behavior: the higher the searching behavior of the public in a network space, the higher the attention of the public on event, and the higher the risk perception [45]. The dynamic data regarding these searches are obtained from Baidu or Google searching indexes. The second method is based on negative emotions online. The public expression of negative emotions is closely correlated with the risk perception at the psychological level: the higher the negative emotion, the higher the risk perception [46]. Here, the dynamic data are sourced from the information posted by the public on the Internet (tapped from big data texts), where emotion lexicon technology is used to analyze emotions within information [47]. In this paper, the first method was used to acquire the big data concerning public risk perception. 

Most western scholars used Google search to identify the risk perception in their studies. For example, Da et al. (2011) [48] measured the search volume of 19 keywords related to stock names in 2011 using the Google search engine, and then analyzed the keywords with Russell 3000 indexes in 2004–2008, and finally obtained the result that search volume is positively correlated with the short-term yield of the Russell index, but is negatively correlated with the long-term yield, signifying that the searching behavior of the public using keywords related to stocks represents their perception of financial and economic risks. Moreover, certain scholars also used search behaviors to analyze the relationships between the macro-economic index and consumer confidence index, and the predicating effect of keyword search volume on financial markets [49]. The research results showed that the greater the number of negative words searched for by the public, the lower their confidence in the financial market. 

Chinese scholars mainly used the Baidu index to carry out their research. The keywords used on Baidu reflect the search demands of the public (netizens). The Baidu Corporation computes the search change rate of keywords using a sorting algorithm, thereby forming the Baidu index. Currently, the studies on risk perception using Baidu indexes in China concentrate on two fields. The first is the relation between the Baidu indexes and economic and financial risks; for example, Zhang et al. (2014) [50] used the Baidu indexes to measure the attention of users on stocks, and then measured the attention of common investors, and finally analyzed the influencing mechanism of common investors’ attention on stock liquidity and stock yield. The authors found that the Baidu indexes reflected the common investors’ perception of stock risks. The second is the public opinion related to risk perception of an event. Chen and Lin (2013) [51] used the Baidu indexes to research the spatial–temporal evolution of online public opinion and analyze the changing characteristics of the Baidu indexes during the online events of “Xiao Yueyue”, “Guo Meimei”, and “Yao Jiaxin” in the spatial–temporal dimension of attention. The results showed that the Baidu index reflected the changing situations of online public opinion about emergency events at a comparatively effective level, namely, the Baidu index could reflect the evolution of online opinion on a risk event. 

The main hypothesis of the current study is that the more the public search for or pay attention to certain content, the stronger the perceived uncertainty of the event, and the higher the risk perception. Certain scholars adopted the questionnaire approach to verify the hypothesis, i.e., by issuing 400 questionnaires in a region where a certain policy was executed. The results showed that the regression coefficient between the public risk perception and the information search behavior of the public was 0.8 (*p* < 0.001), so the higher the respondents’ perception level of the possible risk aroused by a policy, the larger the hazard of risk, and the more active the information search behavior [52]. 

### 2.3. Prediction of Risk Perception

There are currently few studies that focus on the prediction of risk perception. When summarizing the existing research on the topic, the findings revealed that scholars reached consensus in two main areas. (1) The prediction model can be established using risk perception’s influence factors (for example, van der Linden (2015) [53]) on the basis of climate change risks, thus establishing the climate change risk perception model (CCRPM) by adopting cognitive factors (related scientific knowledge), related experience (emotional assessment and personal experience), sociocultural influence (culture, view of value, world view, and social common cognition) and sociodemographic characteristics (sex, political ideology, educational level, age, income) as the main predictive factors to measure the public’s risk perception. There are many factors influencing public risk perception, and these factors not only affect risk perception independently, but also affect risk perception under cross action. Hence, how to establish an effective predictive model is the problem to be solved in this paper. (2) An effective potential method in predictive analysis is the back propagation (BP) neural network method; for example, Zhang et al. (2021) [54], on the basis of droughts, predicted the risk perception of farmers using the BP neural network method by adopting the disastrous situation/environment, farmers’ economic income, and the planting structure of crops as the main predictive factors. In this paper, we explored the use of such a method to predict risk perception.

## 3. Data and Research Methods

**Figure 1:**
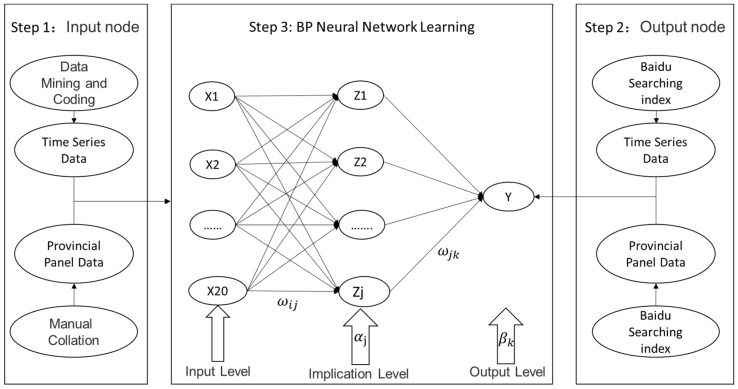
Research procedures.

A back propagation (BP) neural network is a type of machine learning. It is based on a gradient descent strategy to regulate the weight and the threshold value of a network connection using counter propagation, until the error between the predicted value and the actual value reduces to an acceptable range or to the preset number of learning iterations. In this paper, we selected the three-layer BP neural network of a single implication level to predict public risk perception, including input level, implication level, and output level. The studies in this paper were carried out by determining the input node and output node, and using BP neural network learning; see Figure 1 for the research procedures.

### 3.1. Acquisition of Input Node

Through the review of the literature concerning the influence factors of risk perception, we found that internal characteristics such as demographic characteristics, risk experience, and economic characteristics, and external characteristics such as crisis information and risk characteristics may have an influence on risk perception. Therefore, in this paper, we acquired the input node data from two aspects, namely, internal characteristics and external characteristics. Our research data originated from China’s Internet. From 2018 to the present, the topical issues with a significant influence on Chinese people were the “Sino–US trade friction” and “COVID-19 pandemic”. The “Sino–US Trade Friction” event is a representative case of economic environment risk, whereas the “COVID-19 pandemic” is a representative case of public health risk.

Firstly, the time series data were acquired. The Sino–US trade friction has attracted the attention of the public in China since March 2018 and has continued since. Attention on this event gradually declined from the end of October 2019, when Chinese and American leaders both announced that the negotiations had achieved substantive results. On the 21 January 2020, the first news briefing for the COVID-19 pandemic in China was held by the Guangdong Information Office and Nanshan Zhong, a famous scientist in China, announced that the virus can spread from person to person, the event of the COVID-19 pandemic has attracted the attention of the public in China. By the end of August 2021, China had entered a state whereby the epidemic prevention policy had matured, and while the epidemic erupted occasionally in various places, the overall situation was mainly stable. As shown in Figure 1, we first obtained the time series data by data mining and encoding, where the sample time for the Sino–US trade friction event was from 1 March 2018 to 31 October 2019, and the sample time of the COVID-19 pandemic event was from 21 January 2020 to 31 August 2020.

Secondly, the provincial panel data were acquired. We organized the provincial panel data from the Chinese mainland taken from the National Statistics Bureau of China, and finally combined the time series data and the provincial panel data. 

Lastly, the time series data and provincial panel data were combined. Except for the missing data, we finally obtained 16,005 effective data related to the “Sino–US trade friction” event and 18,259 effective data related to the “COVID-19 pandemic” event for conducting our analysis. Table 1 shows the seven influence factors and 20 measurable variables of risk perception. All measurable variables were used as the input nodes. In the paper, the input node number of the BP neural network, as constructed herein, was 20.

#### 3.1.1. Internal Characteristic Index

According to the review of the literature, three factors, namely, demographic characteristics, risk experience, and experience characteristics, were selected as the predictive variables of public risk perception. The demographic characteristics were measured by the total population (X1) and the sex ratio (X2) of a certain province.

The risk experience was measured according to the regional distribution of risks in China. According to the research results, the financial crisis risks in the eastern region and the middle region of China were higher than those in the western region [55], but the natural disaster risks in the western and middle regions of China were higher than those in the eastern region [56]. Thus, in this paper, we set the provinces in the eastern region as “1” under “Financial risk experience (X3)”, the provinces in the middle region as “1” under “Compound risk experience (X4)”, and the provinces in the western region as “1” under “Natural disaster risk experience (X5)”.

The economic characteristics were taken from the cultural and economic characteristics provided in the literature review. The cultural characteristics are rather abstract and are hard to use as quantitative indexes; most scholars have verified that there is a significant relation between culture and economy [57,58,59], and therefore in this paper we selected economic characteristics as the predictive factor of risk perception, and used four variables: GDP (X6), per capita GDP (X7), foreign trade amount (total export–import volume of local area in which it runs, X8) and domestic trade amount (total retail sales of consumer goods, X9) to make our measurement.

#### 3.1.2. External Environment Indexes

According to the literature review, three factors, namely, media intervention, government intervention, and risk characteristics, were selected as the predictive variables of public risk perception. The data in this paper are excerpted from Internet big data, so media intervention is affected by a province’s Internet popularity rate and the media’s reporting of the Sino–US trade friction or COVID-19 pandemic. Thus, we selected Internet popularity (X10) and the Baidu media index (X11) as the measurement variables of media intervention, whereby the Baidu media index is the publicity of an issue reported by the media (computed by the Baidu Corporation, http://index.baidu.com (accessed on 1 June 2022)).

Studies on government intervention behaviors are based on the “Meaning Making Theory of Crisis Government” [50]. According to the theory, the government constructs the public’s perception of a crisis using three strategies: rituals, masking, and framing. Rituals refers to the meaning-making of a government, which can be represented by the actions of officials or officers, such as standing in silent tribute or mourning, consoling victims, or official investigations into an event [60], in which official channels and leaders play important role [61]. Therefore, ritual coding refers to two variables: whether information is posted on an official website (X12) and whether information about the leader is available (X13). Masking is the practice adopted by governments to weaken the influence of crises that involve deep-rooted conflict and social vulnerability [15]. For the masking strategy, we set the information-weakening indicator (X14). Framing refers to refining certain aspects of a crisis and showing them to audiences through artificial screening [62]; in accordance with the research of Eldridge (2008) [63], governments may use four frames, namely, benefit (X15), emotion (X16), responsibility (X17), and threat (X18), in risk events, so we set the indicators X15 and X18 in this paper. The combined time series and provincial panel data pertaining to government intervention behavior were obtained in two ways. X12/13/15/16/17/18 were obtained using the big data mining of government information released on the Internet, finding the information released by the government on a certain day and carrying out data cleaning and manual encoding (if there was no government information obtained on a certain day, all of the data from that day were treated as missing). X14 was calculated using the normalization result of the government information release data and subtracting the normalization result of the Baidu media index. If the result was less than 0, this signified that the government was weakened relative to media exposure, so the variable code was 1, and the others were 0.

For the risk characteristics, we selected the risk time characteristics as set variables. Within the period under study, the Sino–US trade friction went through the following six stages: experience friction (S1: 1 March 2018–2 May 2018), negotiation (S2: 3 May 2018–14 June 2018), friction (S3, 15 June 2018–1 December 2018), negotiation (S4: 2 December 2018–2 May 2019), friction (S5: 3 May 2018–20 September 2019), and negotiation (S6: 21 August 2019–31 October 2019). At times during stages S1, S3, and S5, the X19 code is 1, and the others are 0; at times during stages S2, S4, and S6, the X20 code is 1, and the others are 0. Within the period under investigation, the COVID-19 pandemic in China went through the following four stages according to a white paper titled “China’s actions to fight the COVID-19 pandemic” published by *Xinhua News*: outbreak period (20 January 2020–20 February 2020), spread control period (21 February 2020–17 March 2020), phased victory period (18 March 2020–28 April 2020), and regular epidemic period (29 April 2020–31 August 2021).During the outbreak period and spread control period, the COVID-19 pandemic aroused some panic among members of the public and was responded to differently, so at these times, the X19 code is 1, and the others are 0. During the phased victory period and regular epidemic period, in which the COVID-19 pandemic returned to normal and all parties reduced their concerns about it, the X20 code is 1, and the others are 0.

### 3.2. Acquisition of Output Node

**Figure 2:**
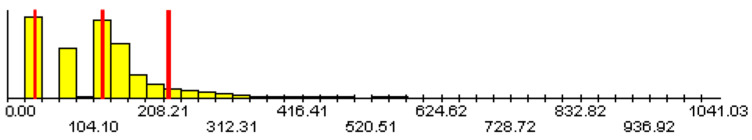
Classification of the public risk perception of the “Sino–US trade friction” (The red lines are the cut-off lines).

**Figure 3:**
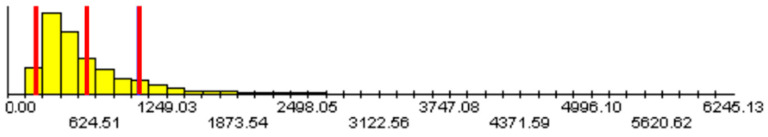
Classification of the public risk perception of the “COVID-19 pandemic” (The red lines are the cut-off lines).

The output node of the BP neural network constructed in this paper is 1. It is the data of public risk perception. By selecting the studied time period from 1 March 2018 to 31 October 2019, obtaining the provincial Baidu indexes (http://index.baidu.com (accessed on 1 June 2022)) of the keywords “Sino–US trade war”, “Sino–US trade friction”, totaling them up to reflect the public perception in local regions for the risks caused by the Sino–US trade friction, and then combining the time series data and provincial panel data, we obtained the output data. Risk perception is a continuous variable, so in order to reflect the high or low level of risk perception, we converted it into a classified variable, and then used the visible discretization function of the SPSS software, and selected the mean and standard deviation based on the specimens to set the cut-off line (see Figure 2). The SPSS software classifies risk perception data into four categories. We then set them as 1–4, from low to high, meaning that the larger the value, the higher the risk perception. Using the same method to analyze, summarize, and categorize the Baidu index of the vocabularies used by the public for “COVID-19” during the period from 21 January 2020 to 31 August 2020, we were able to classify the public risk perception in China for the COVID-19 pandemic (see Figure 3).

### 3.3. Learning of BP Neural Network

Figure 2 shows the BP neural network structure, where the input level sample is $\;X_{i}$, the output of the implication level is $Z_{j}$, and the output of the output level is $\;Y_{k}$. The weight between the input level and implication level is *ω_ij_*, and the weight between the implication level and output level is *ω_jk_*. The threshold values of the implication level and the output level are respectively $\;\alpha_{j}$ and *β_k_*. The standard learning procedures of the BP neural network are described in the following subsections [64].

#### 3.3.1. Calculation of Node Number of Implication Level

As shown in Figure 1, the quantity of the input node and output node was respectively determined in Step 1 and Step 2, so when proceeding to Step 3, we first needed to determine the quantity of the implication level node. If the node is too large, it may result in the network becoming complicated, or may even cause overfitting. If the node is too small, it may result in misconvergence. Presently, there is no ideal analytic formula to determine the optimal node number for the implication level. Most scholars adopted Formula (1) to obtain the implication level node value. In this paper, we also adopted the formula to compute the node number. Here,  m is the node number of the input level, which is 20 in this paper;  n  is the node number of the output level, which is 1 in this paper;  γ is the constant number from 1 to 10; and N is the node number of the implication level.

(1) $$\;N=\sqrt{m+n}+\gamma$$

The most common method used to calculate the value of  γ is to do the tests one by one for  γ according to Formula (1), compare the different node numbers of  N of the implication level and the different mean square errors of the network training, and then judge and select the optimal implication level node [65]. The specific procedures are: (1) randomly initialize the weight $\;\omega_{ij}\;$ between the input level and implication level, the weight $\;\omega_{jk}$ between the implication level and output level, and the threshold value, namely, $\;\alpha_{j}$ and $\;\beta_{k}$, of the implication level and output level into a value from −1 to +1; (2) compute the output $\;Z_{j}$ of the implication level according to Formula (2), and the output $\;Y_{k}$ of the output level according to Formula (3), where the f in Formula (2) is the tangential Sigmoid function: $f\left(x\right)=\frac{e^{x}-e^{-x}}{e^{x}+e^{-x}}$. The f in Formula (3) is the logarithmic Sigmoid function: $\;f\left(x\right)=\frac{1}{1+e^{-x}}$; (3) compute the mean square error (E) according to the error function of Formula (4), where Y is the actual value and $\;Y^{*}$ is the predicted value (in order to correspond to the actual value, the value is rounded off). In this paper, the actual value is the public risk perception data. Through training, the neural network’s predicted value approached the actual value as closely as possible, namely, the error  E tended to be low, signifying better model performance. In the paper, the target error was set to 0.005, and the initial threshold value was 0. (4) Test γ=[1,10], respectively, and test every γ value ten times to obtain E, and then calculate the mean value by removing the maximum value and the second largest value, comparing the mean value of E in different γ values, and finally putting the γ value (when the value is lowest) into Formula (1) to calculate the node number of the implication level.

(2) $$Z_{j}=f\left(\sum_{i=1}^{m}\omega_{ij}X_{i}+\alpha_{j}\right),j=1,\;2,\;\ldots ,N$$

(3) $$Y_{k}=f\left(\sum_{i=1}^{N}\omega_{jk}Z_{i}+\beta_{k}\right),\;k=1$$

(4) $$E=\frac{1}{2}{\left(Y^{*}-Y\right)}^{2}$$

#### 3.3.2. Performance Test of BP Neural Network

Currently, $R^{2}$ is generally used to judge the effectiveness of a BP neural network. The specific procedures are: (1) after determining the node number of the implication level, update the weight $\omega_{ij}$ from the input level to the implication level, and the weight $\omega_{jk}$ from the implication level to the output level according to Formulas (5) and (6), where θ is the learning rate. For the learning rate, the larger the learning rate is, the quicker the training speed is. However, if it is too large, it will affect the stability of the network. Thus, the learning rate is usually valued between 0.01 and 0.8 [65]; in this paper, it was 0.01. (2) Update the threshold values $\alpha_{j}$ and $\beta_{k}$ of the implication level and output level according to Formulas (7) and (8). (3) By training on the Internet, make the actual output approach the expected output as much as possible, until the maximum training frequency is reached or the error precision requirement is met. Finally use Formula (9) to compute the determination coefficient $\;R^{2}$, where $Y_{i}^{*}$ (i = 1, 2, …, M) is the predicted value of the i  specimen; $Y_{i}$(i = 1, 2, …, M) is the actual value of the i specimen; M is the number of specimens under testing; and l is the number of specimens under training. The closer the $\;R^{2}$ approaches 1, the better the prediction result of network.


(5) $$\omega_{ij}=\omega_{ij}+\theta Z_{j}\left(1-Z_{j}\right)X_{i}\sum_{k=1}^{3}\omega_{jk}E_{k}$$

(6) $$\omega_{jk}=\omega_{jk}+\theta E_{k}$$

(7) $$\alpha_{j}=\alpha_{j}+\theta Z_{j}\left(1-Z_{j}\right)\sum_{k=1}^{3}\omega_{jk}E_{k}$$

(8) $$\beta_{k}=\beta_{k}+\theta E_{k}$$

(9) $$R^{2}=\frac{{\left(l\sum_{i=1}^{l}Y_{i}^{*}Y_{i}-\sum_{i=1}^{l}Y_{i}^{*}\sum_{i=1}^{l}Y_{i}\right)}^{2}}{\left(l\sum_{i=1}^{l}Y_{i}^{*}{}^{2}-{\left(\sum_{i=1}^{l}Y_{i}^{*}\right)}^{2}\right)\left(l\sum_{i=1}^{l}Y_{i}{}^{2}-{\left(\sum_{i=1}^{l}Y_{i}\right)}^{2}\right)}$$

#### 3.3.3. Computation of Every Input Node Weight

In order to explore the prediction of every influence factor on public risk perception in the BP neural network, we also computed the influence weight of every input index for public risk perception in this paper. Use the normalization result $\omega_{i}$ of the total sum of the absolute values from input index $X_{i}$ to all implication level nodes as the influence weight, and then use Formula (10) to compute.


(10) $$\omega_{i}=\frac{\sum_{j=1}^{N}|\omega_{ij}|}{\sum_{i=1}^{m}\sum_{j=1}^{N}\left|\omega_{ij}\right|}\;i=1,\;2,\;\ldots ,\;m;\;j=1,\;2,\;\ldots ,N$$

## 4. Research Results

### 4.1. Calculation Results of Implication Level Nodes

**Figure 4:**
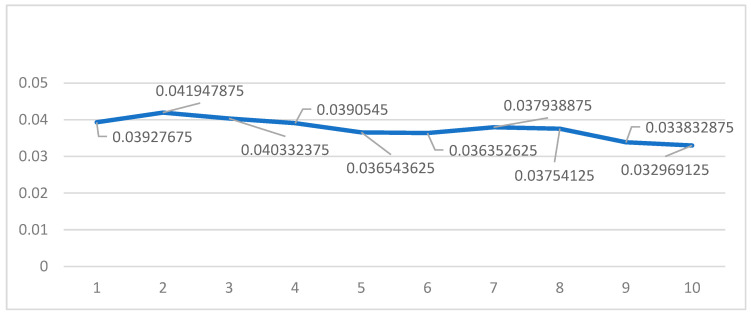
Mean value of MSE at different values of γ for “Sino–US trade friction”.

In the case of Sino–US trade friction, we used 15,978 data from 1 March 2018 to 30 October 2019 as the training specimen, and 27 data (test data from four provinces, namely, Gansu, Ningxia, Qinghai, and Xinjiang were missing on this day, since there was no government intervention information in those provinces on that specific day) from 31 October 2019 as the test specimen. Firstly, we set the initial node number of the implication level to 6(γ = 1), and then we trained 10 times. After removing the largest and second largest errors, we calculated the mean value of the last eight errors. Finally, we set the node number of the implication level as a value from 6 to 15. Table 2 shows the mean square error at different implication level nodes. Thus, we obtained the mean error of the network training at different implication level node numbers (see Figure 4). According to Figure 4, we can see that as the γ value increases, the error of mean square of BP neural network tends to decline, but certain values bounce up in the process; when γ is 2 and 7, the error is at its maximum value; when γ is 10, the error is at its minimum value, so the implication level node of network in this case was selected as 15.

**Figure 5:**
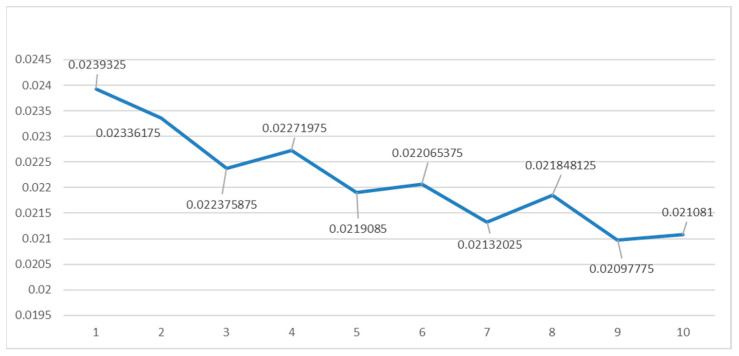
Mean value of MSE at different values of γ for “COVID-19 pandemic”.

In the case of the COVID-19 pandemic, we used 18,228 data from 21 January 2020 to 30 August 2020 as the training specimen, and 31 data from 31 October 2019 as the test specimen. We used the same method to test the values of γ from 1 to 10 (see Table 3 and Figure 5 for results), so the implication level node of the network in this case was selected as 14.

### 4.2. Performance Test Results of BP Model

**Figure 6:**
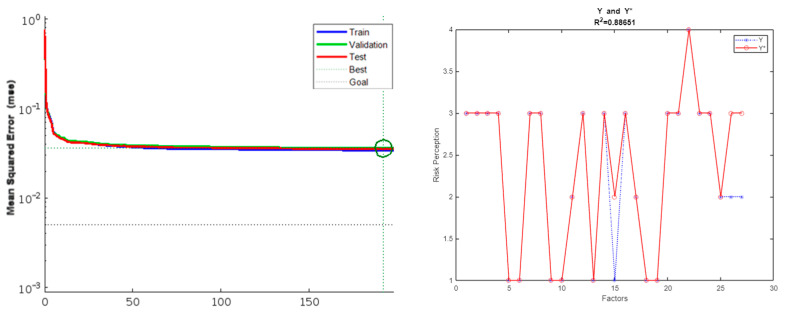
MSE and $R^{2}$ of the model for “Sino–US trade friction”.

**Figure 7:**
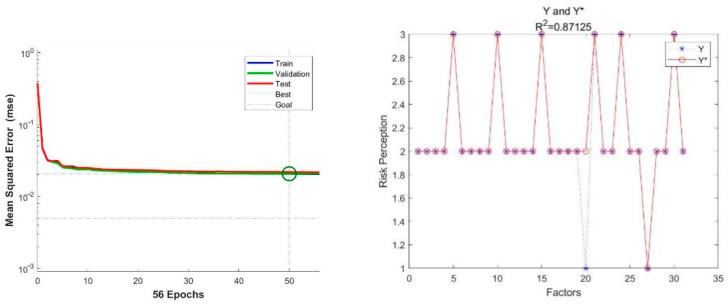
MSE and $R^{2}$ of the model for “COVID-19 pandemic”.

Figure 6 shows the least mean square error of the BP neural network model for “Sino–US trade friction”, as constructed in this paper, which was 0.036073 (at the epoch 192). Figure 6 also shows the $R^{2}$ of the model, which was 0.88651. Figure 7 shows the least mean square error of the BP neural network model for “COVID-19 pandemic”, which was 0.020629 (at epoch 50). Figure 7 also shows the $R^{2}$ of the model, which was 0.87125. The results showed that the BP neural network model exhibited good performance in both cases.

For the difference in the predicted value and actual value in those two cases, see Table 4 and Table 5. Among the risk perception predictions for “Sino–US trade friction” in 27 areas from the Chinese mainland on 31 October 2019, 24 of which were the prediction preparations where the prediction in three areas, namely, Shandong, Tibet, and Shaanxi, errors occurred: the error was less than Level 1, and all predictions of risk perception were high class 1. Among the risk perception predictions for the COVID-19 pandemic in 31 areas of the Chinese mainland on 31 October 2019, 30 of which were the prediction preparations, errors only occurred in the prediction for Qinghai.

### 4.3. Determining the Weight of Every Prediction Index

#### 4.3.1. Index Weight in the Sino–US Trade Friction

**Figure 8:**
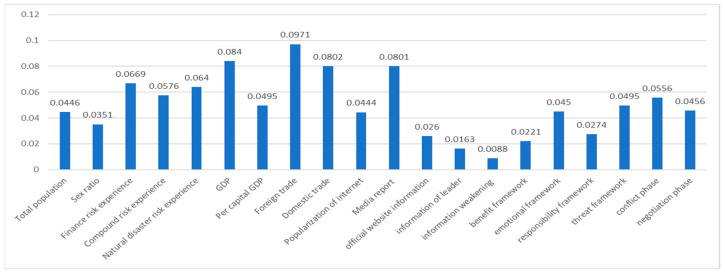
Weight of every prediction index in “Sino–US trade friction”.

In the case of the “Sino–US trade friction”, a weight of 20 indices was computed according to Formula (10): W = (0.0446, 0.0351, 0.0669, 0.0576, 0.0640, 0.0840, 0.0495, 0.0971, 0.0802, 0.0444, 0.0801, 0.0260, 0.0163, 0.0088, 0.0221, 0.0450, 0.0274, 0.0495, 0.0556, 0.0456); the histogram is shown in Figure 8.

As shown in Figure 8, in the case of the Sino–US trade friction, among internal characteristic indexes, the weight of economic characteristics (the mean value of X6–X9 was 0.0777) for risk perception prediction was the highest, and among these, the weight of the prediction for the foreign trade variable (X8) was the highest, which was related to the foreign trade events related to the Sino–US trade friction. The weight of risk experience (the mean value of X3, X4, and X5 was 0.062833) was second highest, and the weight of the prediction for financial risk experience (X3) was the highest of these, which meets the characteristics of financial risk. However, the weight of the demographic characteristics (the mean value of X1 and X2 was 0.03985) was the lowest.

Among the external environmental indexes, the weight of media intervention (the mean value of X10 and X11 was 0.06225) was the highest. Compared with the popularity of the Internet (X10), the weight of the prediction for media reporting (X11) was higher. The mean weight of risk characteristics (X19 and X20) was 0.0506, where the prediction variable at the phase of conflict (X19) was higher, which meets the research result; i.e., the risk perception at the period of high risk was higher, as described in the literature review. The weight of government intervention (X12–X18) was lowest with a mean value of 0.027871, and the weights for the prediction for emotion framework (X16) and threat framework (X18) were higher.

#### 4.3.2. Index Weight in the COVID-19 Pandemic

**Figure 9:**
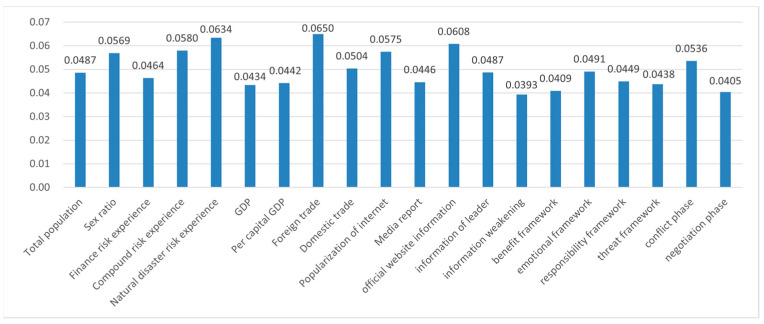
Weight of every prediction index for “COVID-19 pandemic”.

In the case of the COVID-19 pandemic, the weights of 20 indices were computed according to Formula (10): W= (0.0487, 0.0569, 0.0464,0.0580, 0.0634, 0.0434, 0.0442,0.0650, 0.0504, 0.0575, 0.0446, 0.0608, 0.0487, 0.0393, 0.0409, 0.0491, 0.0449,0.0438, 0.0536, 0.0405); the histogram is shown in Figure 9.

As shown in Figure 9, the weight of risk experience (the mean value of X3, X4, and X5 was 0.05593) in the case of the COVID-19 pandemic, different from the highest weight of economic characteristics for the Sino–US trade friction, was the highest among the internal characteristics indicators, among which the predictive weight (X5) of natural disaster risk experience was the highest. Following this was the weight of the demographic characteristics (the mean value of X1 and X2 was 0.0528); the predictive weight of the economic characteristics (the mean value of X6–X9 was 0.05075) was the lowest. Compared with the case of the Sino–US trade friction, there was no huge difference in the weights of the various indicators of the internal characteristics in the case of the COVID-19 pandemic.

Among the environmental indicators, the weight of media intervention (the mean value of X10 and X11) was the highest; compared with media reporting (X11), the predictive weight of Internet popularity (X10) was higher, but the weight of media reporting in the case of the Sino–US trade friction was higher. The second-highest weight ranking was risk characteristics (X19 and X20) with an average weight of 0.04705, among which the predictor variable at the phase of conflict (X19) was higher, similar to the result in the case of the Sino–US trade friction. The weight of government intervention (X12–X18) was the lowest, averaging 0.04037, among which the predictive weights of information source on official websites (X12) and emotional framework (X16) were higher, especially for the information source on official websites, for which the predictive weight was as high as 0.0608. This result was significantly different from the case of Sino–US trade friction, signifying that the authority of the information source for the COVID-19 pandemic influenced public perception to a high degree.

## 5. Conclusions

### 5.1. Major Findings and Contributions

#### 5.1.1. Influence Factors can Effectively Predict Public Risk Perception of Topical Issues

Through a literature review, we found that the internal characteristics of groups, such as demographic characteristics, economic characteristics, and risk experience, can affect public risk perception, whereas external environment factors such as crisis information and risk characteristics can also have an effect; however, there is currently a dearth of literature on the prediction of public risk perception. In this paper, the empirical analysis of two cases, namely, the “Sino–US trade friction” and the “COVID-19 pandemic”, was conducted using the BP neural network method (machine learning). The main finding is that influence factors, such as internal characteristics and external environment characteristics, can effectively predict public risk perception (the $R^{2}$ of predictive models in the two cases were respectively 0.88651 and 0.87125, with the predictive result being better).

This paper makes two theoretical contributions, as follows. 1. The empirical study of two topical issues, namely, the “Sino–US trade friction” and the “COVID-19 pandemic”, was added to the research on public risk perception. 2. The research field of the prediction of public risk perception of topical issues was further enriched. This paper is significant in that it can serve to inform government practice in terms of the control of public opinion and the evaluation of social risk. All of the predictive variables in this paper were easily acquired, and were significant in establishing a predictive model of public risk perception of topical issues based on big data analysis, machine learning methods, etc., and can be incorporated into the practices of governmental and third-party departments.

#### 5.1.2. External Environment Can Effectively Lead Public Risk Perception of Topical Issues

As there are so few existing studies on the prediction of public risk perception, there is less of a focus on the rise and fall of public risk perception associated with adjustments to the predictor variables. However, with topical social issues, effective guidance regarding public risk perception is crucial. I addition, in the two cases in this paper, we computed the influence weight of every predictor variable for public risk perception. Through these weights, we can identify ways of guiding public risk perception. Since internal characteristics are usually inherent, media intervention and government intervention in external environment factors can be adjusted by risk managers; thus, another finding in this paper is that public risk perception can be effectively led by adjusting the external environment.

This finding has three theoretical contributions. 1. The weight of media intervention may be higher than that of government intervention; such a conclusion was well verified in the two cases studied in this paper, so the conclusion is that it is necessary to adjust risk perception via the media when there is a topical issue. 2. The predictive weight of topical issues at the conflict phase was higher than at the cooling-off phase; such a conclusion was also well verified in this study. This enlightens our practice, namely, special attention should be paid to the guidance of public risk perception at the point in time when high risk occurs. 3. In the case of “Sino–US trade friction”, the weight of media reporting in media intervention was higher than that of Internet popularity, contrary to the case of the ”COVID-19 pandemic”; in “Sino–US Trade Friction”, the predictive weight of the emotion frame used for government intervention was the highest, but the predictive weight of information source on the official website in the case of the “COVID-19 pandemic” was highest. The possible reason for this may be that the Sino–US trade friction is a risk to the economic environment, and the people who pay more attention to this topic might belong to highly educated groups with high exposure to media reporting, so they are more easily affected by governmental emotion; however, the COVID-19 pandemic is a public health risk that affects all people, so public risk perception may firstly be influenced by their exposure to Internet, and secondly by the authority of the information source to which they have access. Hence, the implication for practice is to set guidance variables against different types of risk events.

### 5.2. Limitations and Suggestions for Future Research

The limitations of the research in this paper are that only two cases were used to verify the predictive model in China’s practice. The two cases, namely, the “Sino–US trade friction” and the “COVID-19 pandemic”, are significantly representative and have received extensive attention in the fields of economic environment risk and public health risk, and are two risk events that have produced far-reaching influences on China. Thus, they are highly representative and appropriate for research on the prediction of public risk perception in China. Considerations for future research include the addition of cases other than China, as well as other types of risk events, to further verify the model and enable comparative study.

## Figures and Tables

**Table 1:** Input vector of BP neural network.

Predictor Variable	Measurable Variable/Input Node	Source	Input Value of Index
Internal characteristics			
Demographic characteristics	X1 Total population	National Bureau of Statistics of China	Value
	X2 Sex ratio (male/female)	National Bureau of Statistics of China	Value
Risk experience	X3 Finance risk experience	More financial crisis experience, less natural disaster experience	0.1
	X4 Compound risk experience	More financial crisis and natural disaster experience	0.1
	X5 Natural disaster risk experience	More natural disaster experience, less financial crisis experience	0.1
Economic characteristics	X6 GDP	National Bureau of Statistics of China	Value
	X7 Per capital GDP	National Bureau of Statistics of China	Value
	X8 Foreign trade	National Bureau of Statistics of China	Value
	X9 Domestic trade	National Bureau of Statistics of China	Value
External environment			
Media intervention	X10 Popularization of Internet	Statistical Reports on Internet Development in China, http://www.cnnic.net.cn/ (accessed on 1 June 2022)	Value
	X11 Media report	Baidu Media index; http://index.baidu.com (accessed on 1 June 2022)	Value
Government intervention	X12 Posts information on official website or not	Manual encoding	0.1
	X13 Provides information about leader or not	Manual encoding	0.1
	X14 Uses information weakening strategy or not	Manual encoding	0.1
	X15 Uses the benefit frame or not	Manual encoding	0.1
	X16 Uses the emotion frame or not	Manual encoding	0.1
	X17 Uses the responsibility frame or not	Manual encoding	0.1
	X18 Uses the threat frame or not	Manual encoding	0.1
Risk characteristics	X19 Is in the conflict phase or not	Manual encoding	0.1
	X20 Is in the cooling-off phase or not	Manual encoding	0.1

**Table 2:** MSE at different implication level node numbers (*N*) for “Sino–US trade friction”.

*N*	6	7	8	9	10	11	12	13	14	15
1	0.03843	0.041288	0.04026	0.037899	0.036723	0.03695	0.041334	0.041546	0.035998	0.035452
2	0.038797	0.040899	0.040164	0.040001	0.036203	0.034511	0.038502	0.039368	0.033054	0.031881
3	0.037166	0.042028	0.041301	0.037746	0.036064	0.036079	0.03839	0.03994	0.033773	0.032317
4	0.040197	0.043781	0.038962	0.038004	0.038816	0.03644	0.038882	0.035211	0.035942	0.033913
5	0.038399	0.042905	0.039712	0.037565	0.03704	0.036506	0.037156	0.037334	0.03319	0.0336
6	0.039094	0.041749	0.042155	0.043736	0.037535	0.03787	0.035949	0.036542	0.032057	0.033272
7	0.040387	0.041865	0.039723	0.040224	0.034747	0.038156	0.039725	0.037975	0.035863	0.033655
8	0.042235	0.043282	0.041622	0.042796	0.037872	0.036668	0.037521	0.038288	0.034709	0.032697
9	0.041834	0.042483	0.041492	0.041177	0.036453	0.036274	0.037597	0.037562	0.033135	0.032562
10	0.041744	0.042366	0.041045	0.03982	0.037584	0.037393	0.039514	0.03805	0.034882	0.033769
S	0.041834	0.043282	0.041622	0.042796	0.037872	0.03787	0.039725	0.03994	0.035942	0.033913
M	0.042235	0.043781	0.042155	0.043736	0.038816	0.038156	0.041334	0.041546	0.035998	0.035452
A	0.039277	0.041948	0.040332	0.039055	0.036544	0.036353	0.037939	0.037541	0.033833	0.032969

S = second largest value; M = maximum value; A = average without second largest and maximum value.

**Table 3:** MSE at different implication level node numbers (*N*) for “COVID-19 pandemic”.

*N*	6	7	8	9	10	11	12	13	14	15
1	0.024931	0.023911	0.022735	0.023191	0.02281	0.022264	0.021038	0.021993	0.022286	0.02226
2	0.024628	0.023652	0.024554	0.021627	0.021808	0.022545	0.021293	0.023511	0.021676	0.021454
3	0.024593	0.024658	0.022684	0.022838	0.021443	0.024211	0.022768	0.021613	0.020995	0.022321
4	0.024709	0.023674	0.021766	0.021945	0.021509	0.022157	0.022252	0.021199	0.021362	0.020565
5	0.023643	0.022454	0.021477	0.023813	0.023024	0.022271	0.021368	0.022018	0.021549	0.021106
6	0.022591	0.023708	0.022449	0.024008	0.022284	0.022097	0.022242	0.022135	0.021545	0.021935
7	0.026123	0.022861	0.022823	0.022809	0.022792	0.022608	0.020362	0.020633	0.021154	0.02112
8	0.023222	0.024408	0.022363	0.024112	0.021709	0.022643	0.02243	0.023532	0.019564	0.022017
9	0.024576	0.022226	0.02271	0.023301	0.023679	0.02156	0.020604	0.022579	0.020798	0.019582
10	0.023498	0.025131	0.024482	0.022234	0.020913	0.021021	0.021403	0.022615	0.020855	0.020869
S	0.024931	0.024658	0.024482	0.024008	0.023024	0.022643	0.02243	0.023511	0.021676	0.02226
M	0.026123	0.025131	0.024554	0.024112	0.023679	0.024211	0.022768	0.023532	0.022286	0.022321
A	0.023933	0.023362	0.022376	0.02272	0.021909	0.022065	0.02132	0.021848	0.020978	0.021081

S = second largest value; M = maximum value; A = average without second largest and maximum value.

**Table 4:** Comparison of actual values and predicted values for “Sino–US trade friction”.

Time	Location	Actual Value	Predicted Value	Time	Location	Actual Value	Predicted Value
31 October 2019	Beijing	3	3	31 October 2019	Jiangxi	3	3
31 October 2019	Tianjin	3	3	31 October 2019	Shandong	1	2
31 October 2019	Hebei	3	3	31 October 2019	Henan	3	3
31 October 2019	Shanxi	3	3	31 October 2019	Hubei	2	2
31 October 2019	Inner Mongolia	1	1	31 October 2019	Hunan	1	1
31 October 2019	Liaoning	1	1	31 October 2019	Guangdong	1	1
31 October 2019	Jilin	3	3	31 October 2019	Guangxi	3	3
31 October 2019	Heilongjiang	3	3	31 October 2019	Hainan	3	3
31 October 2019	Shanghai	1	1	31 October 2019	Chongqing	4	4
31 October 2019	Jiangsu	1	1	31 October 2019	Sichuan	3	3
31 October 2019	Zhejiang	2	2	31 October 2019	Guizhou	3	3
31 October 2019	Anhui	3	3	31 October 2019	Yunnan	2	2
31 October 2019	Fujian	1	1	31 October 2019	Tibet	2	3
31 October 2019	Shaanxi	2	3				

**Table 5:** Comparison of actual values and predicted values for “COVID-19 pandemic”.

Time	Location	Actual Value	Predicted Value	Time	Location	Actual Value	Predicted Value
31 August 2021	Anhui	2	2	31 August 2021	Liaoning	2	2
31 August 2021	Beijing	2	2	31 August 2021	Inner Mongolia	2	2
31 August 2021	Fujian	2	2	31 August 2021	Ningxia	2	2
31 August 2021	Gansu	2	2	31 August 2021	Qinghai	1	2
31 August 2021	Guangdong	3	3	31 August 2021	Shandong	3	3
31 August 2021	Guangxi	2	2	31 August 2021	Shanxi	2	2
31 August 2021	Guizhou	2	2	31 August 2021	Shaanxi	2	2
31 August 2021	Hainan	2	2	31 August 2021	Shanghai	3	3
31 August 2021	Hebei	2	2	31 August 2021	Sichuan	2	2
31 August 2021	Henan	3	3	31 August 2021	Tianjin	2	2
31 August 2021	Heilongjiang	2	2	31 August 2021	Tibet	1	1
31 August 2021	Hubei	2	2	31 August 2021	Xinjiang	2	2
31 August 2021	Hunan	2	2	31 August 2021	Yunnan	2	2
31 August 2021	Jilin	2	2	31 August 2021	Zhejiang	3	3
31 August 2021	Jiangsu	3	3	31 August 2021	Chongqing	2	2
31 August 2021	Jiangxi	2	2				

## Data Availability

The dataset generated and analyzed in this study is not publicly available. The dataset is available from the corresponding author on reasonable request.
